# Mathematical modelling of oxygen transport in a muscle-on-chip device

**DOI:** 10.1098/rsfs.2022.0020

**Published:** 2022-08-12

**Authors:** David Hardman, Manh-Louis Nguyen, Stéphanie Descroix, Miguel O. Bernabeu

**Affiliations:** ^1^ Centre for Medical Informatics, Usher Institute, The University of Edinburgh, Edinburgh EH8 9BT, UK; ^2^ The Bayes Centre, The University of Edinburgh, Edinburgh EH8 9BT, UK; ^3^ Institut Curie, Laboratoire Physico Chimie Curie, Institut Pierre-Gilles de Gennes, CNRS UMR168, 75005 Paris, France

**Keywords:** CFD, tissue engineering, hypoxia, muscle

## Abstract

Muscle-on-chip devices aim to recapitulate the physiological characteristics of *in vivo* muscle tissue and so maintaining levels of oxygen transported to cells is essential for cell survival and for providing the normoxic conditions experienced *in vivo*. We use finite-element method numerical modelling to describe oxygen transport and reaction in a proposed three-dimensional muscle-on-chip bioreactor with embedded channels for muscle cells and growth medium. We determine the feasibility of ensuring adequate oxygen for muscle cell survival in a device sealed from external oxygen sources and perfused via medium channels. We investigate the effects of varying elements of the bioreactor design on oxygen transport to optimize muscle tissue yield and maintain normoxic conditions. Successful co-culturing of muscle cells with motor neurons can boost muscle tissue function and so we estimate the maximum density of seeded neurons supported by oxygen concentrations within the bioreactor. We show that an enclosed bioreactor can provide sufficient oxygen for muscle cell survival and growth. We define a more efficient arrangement of muscle and perfusion chambers that can sustain a predicted 50% increase in maximum muscle volume per perfusion vessel. A study of simulated bioreactors provides functions for predicting bioreactor designs with normoxic conditions for any size of perfusion vessel, muscle chamber and distance between chambers.

## 1. Introduction

Organ- and tissue-on-chip technologies are promising tools [1] for generating functional tissues from precursor cells on perfused microdevices. A muscle-on-chip device which recapitulates *in vivo* skeletal muscle functionality [2] has the potential to reduce the requirements for animal and human pharmaceutical trials [3] and allow patient-specific models of muscular diseases for personalized treatments.

As current research aims to capture the physiological characteristics of *in vivo* muscle tissue, it is necessary to replicate *in vivo* conditions as closely as possible. Successful co-culturing with motor neurons will innervate muscle tissue, while co-culturing with endothelial cells will aid the perfusion of nutrients, increase myogenesis and improve tissue contraction [4] and culturing cells in three-dimensional boosts the contractile properties of muscle fibres [5].

To culture viable muscle tissue, oxygen availability is essential for cell survival [6]. Determining and maintaining oxygen levels is, therefore, required to ensure experimental reproducibility, yet oxygen concentration is often ignored or unreported in studies [7].

As the goal of muscle-on-chip design is to replicate *in vivo* conditions, oxygen concentrations should be defined accordingly to ensure cultured cells and tissue experience ‘normoxic’ conditions. Levels of higher oxygen concentrations are considered hyperoxic and lower concentrations considered hyopxic. Here, we define normoxic as the typical oxygen concentrations experienced in muscle tissue *in vivo*. As atmospheric oxygen concentrations are significantly higher than *in vivo* normoxic intracellular conditions, a sealed device is required to prevent atmospheric oxygen from diffusing into the system. Much current muscle and muscular disease research requires studying cell behaviours at specific tissue oxygen levels and so the ability to control oxygen concentrations within the device would also allow the effects of hypoxic and hyperoxic environments on muscle cell and tissue behaviour to be studied.

When culturing tissue, the oxygen requirements of the system will change over time as the initial seeding of precursor cells proliferate, differentiate and mature, causing their oxygen consumption rate to rise. Additionally, as muscle tissue matures and increases in volume it will change the material properties of the substrate, altering the rate at which oxygen can diffuse through the device. Experimental measurement of oxygen gradients is difficult [8], and construction and trialling of prototype devices is costly both in terms of materials required and the time taken to engineer and run. Through numerical modelling, *in silico* experiments can be used to estimate transport of oxygen within the device via advection through media flow, diffusion and cell consumption in a fast and inexpensive manner [9,10].

Here, we apply finite-element method (FEM) modelling to a proposed muscle-on-chip device in which oxygen and nutrients are supplied via a perfusion vessel lined with endothelial cells. We firstly assess whether a bioreactor that is sealed from external oxygen sources outside of the perfusion vessel has the capacity to provide sufficient oxygen concentrations to support the culturing of muscle cells. We then investigate the optimal configuration of the device in terms of size and positioning of perfusion vessels and muscle tissue chambers to maximize muscle tissue yield. From this investigation, we can determine configurations of the bioreactor which are likely to replicate oxygen concentrations found in *in vivo* tissues and which are likely to result in hypoxic or in hyperoxic conditions. Finally, we estimate the maximum concentration of neurons which can be seeded and still provide a sufficient supply of oxygen to ensure the growth of viable muscle tissue. Together, these studies provide guidance for replicating *in vivo* conditions in the fabrication of a muscle-on-chip bioreactor as well as a methodology for future tissue culture research.

## 2. Material and methods

### 2.1. Chip geometry

**Figure 1.**
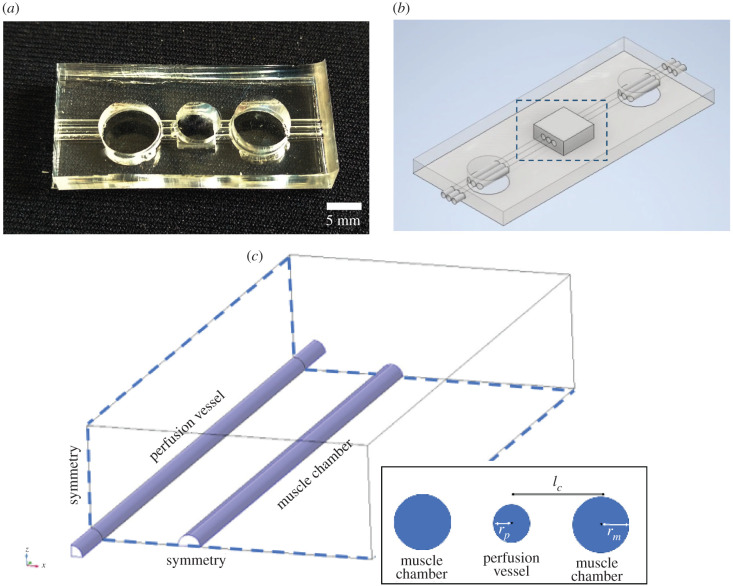
Muscle-on-chip geometry. (*a*) PDMS cast of proposed muscle-on-chip device including peripheral reservoirs, central bioreactor chamber and three inlet and outlet perfusion tubes. (*b*) Overview of chip showing central bioreactor (in dashed box). (*c*) Simulation region of bioreactor showing DMEM filled perfusion vessel (left) and muscle tissue chamber (right) surrounded by collagen I filled chamber with planes of symmetry on base and right-hand side. Inset showing simulation variables; perfusion vessel radius (*r*_*p*_), muscle chamber radius (*r*_*m*_) and distance between channel centrelines (*l*_*c*_).

The proposed muscle-on-chip bioreactor (figure 1*a*,*b*) consists of a central chamber with dimensions 5 mm × 5 mm × 2 mm filled with collagen type I hydrogel from Corning at 6 mg ml^−1^ embedded in PDMS with parallel tubes running through the device. Hollow tube geometry is relevant as *in vivo* skeletal muscle cells, also called myotubes at early stages of development, have elliptical or cylindrical shapes with diameters ranging from a few tens of microns up to hundreds of microns [11]. Tube geometry also mimics the shape of a blood vessel. Vascularization of *in vivo* skeletal muscles arises from arteries with hundreds of microns in diameter, parallel to the muscle axis, which subdivide into arterioles and capillaries penetrating the muscle tissue perpendicularly [12]. Skeletal muscle tissue decellularization studies showed that skeletal muscle cells are embedded in a rich and complex intricated extracellular matrix, mainly composed of collagen type I [13]. Therefore, using collagen type I as the extracellular matrix within our bioreactor is appropriate. This design is composed of three hollow tubes (or channels) embedded in collagen I, with the purpose of hosting three different cell types per channel to mimic the *in vivo* skeletal muscle multicellular environment (muscle cells, endothelial cells, neurons). Peripheral reservoirs are designated to provide nutrients by culture medium diffusion to the central chamber containing muscle cells in case no vascularization is implemented. Initially, the central channel is designated as the muscle cells growth compartment and the two lateral channels are conservatively designated as media perfusion vessels sufficient to provide nutrients/oxygen to the muscle chamber. The two lateral channels are, therefore, seeded with endothelial cells until a lumen is formed, then perfused with Dulbecco’s modified Eagle’s medium (DMEM) (10566016, ThermoFisher Scientific, USA) at a constant rate of flow via an external pump system (Flow EZ, Fluigent company, France) and the central tube is seeded with skeletal muscle progenitor stem cells. Flow in the perfusion tubes is assumed to be fully developed before entering the central chamber and so numerical modelling is applied only to the central chamber (figure 1*c*) with a short inlet and outlet applied to each end of each perfusion tube. The PDMS casing of the proposed bioreactor is permeable to oxygen. In this investigation, we study the effects of sealing the device from atmospheric oxygen, our simulations assume that either the casing or a container surrounding the device is constructed of a material impermeable to oxygen, such as glass.

### 2.2. Governing equations

All numerical modelling of coupled media flow and oxygen transport was performed using COMSOL Multiphysics software. The media within the bioreactor cavity and the muscle tissue chamber remains stationary, while the media moving through the perfusion vessel is assumed to be an incompressible fluid modelled as a steady-state laminar flow with equations,

2.1 ρ(u⋅∇)u=∇⋅[−p+q]

and

2.2 ρ∇⋅u=0.

Where *ρ* is media density, **u** is fluid velocity, *p* is pressure and **q** the viscous diffusion term,

2.3 $$q=\mu (\nabla u+{\left(\nabla u\right)}^{T}),$$

where μ is media viscosity.

The dynamics of oxygen transport within the bioreactor are governed by the following generalized steady state equation,

2.4 Diffusion−Advection−Reactions=0.

Accounting for diffusion, advection in regions with flow and reactions which simulate removal of oxygen via cell metabolism.

Within the perfusion vessel,

2.5 $$D_{\mathrm{DMEM}}\nabla^{2}c-\nabla \cdot uc=0,$$

where *D*_DMEM_ is the coefficient of diffusion in DMEM and *c* is the concentration of oxygen in mol m^−3^. As the medium within the perfusion vessel is flowing,

The endothelial cell lining of the perfusion vessel is assumed to be sufficiently thin to model as a surface reaction, described in the boundary conditions below, and so *R* is set to zero within the vessel. Within the bioreactor cavity there is no media flow and so,

2.6 $$D_{\mathrm{col}}\nabla^{2}c-R_{\mathrm{neur}}=0,$$

where *D*_col_ is the coefficient of diffusion in collagen medium. For simulations without neurons *R*_neur_ = 0, while neuron metabolism is simulated as a constant value dependent on cell density,

2.7 $$R_{\mathrm{neur}}=\rho_{\mathrm{neur}}V_{\mathrm{neur}},$$

where *ρ*_neur_ is density of neurons (cells m^−3^) and *V*_neur_ is the rate of oxygen consumption per neuron. Within the muscle tissue chamber,

2.8 $$D_{m}\nabla^{2}c-R_{\mathrm{MC}}=0,$$

where *D*_*m*_ is the coefficient of diffusion for muscle cells and mature muscle tissue and oxygen consumption is defined as

2.9 $$R_{\mathrm{MC}}=\rho_{m}V_{m},$$

where *ρ*_*m*_ is density of cells per cubic metre. For muscle precursor cells, the rate of oxygen consumption per cell *V*_*m*_ = *V*_*mc*_ is taken as a constant from literature while for mature muscle cells we apply the following Michaelis–Menten reaction,

2.10 $$V_{m}=\frac{V_{M\mathrm{Max}}c}{\left(c+k_{\mathrm{MM}}\right)},$$

where *V*_*M*Max_ is the maximum OCR for mature muscle tissue and *k*_MM_ is the Michaelis–Menten constant for muscle tissue.

### 2.3. Boundary conditions

A table describing the boundary conditions for modelling of flow and mass transport and their algebraic form is included in electronic supplementary material, tables S1 and S2. Flow of DMEM through the perfusion vessel inlet is assumed to be fully developed and time independent with viscosity *μ* = 7.8 × 10^−4^ Pa.s [14]. For all simulations, inlet flow rates were set to provide a wall shear stress of 0.5 Pa independent of perfusion vessel radius (5 × 10^−10^ m^3^ s^−1^ for a tube radius of 100 μm for example), similar to *in vivo* conditions [15]. A no slip boundary condition was applied to vessel walls and pressure was set to zero at the tube outlets.

A constant, uniform oxygen concentration was applied at the inlet and continuity of oxygen concentration flux applied to the boundaries between the perfusion vessel and the collagen filled bioreactor chamber and between the main chamber and the muscle cell chamber. Additionally, the metabolism of oxygen by endothelial cells is simulated as a surface reaction with a Michaelis–Menten kinetic [16],

2.11 $$R_{\mathrm{eth}}=\frac{J_{\mathrm{eth}}c}{c+k_{\mathrm{eth}}M}$$

where *J*_eth_ is the oxygen consumption rate (OCR) flux in endothelial cells and *k*_eth_*M* is the Michaelis–Menten constant for endothelial cells. Coefficients of oxygen permeability through endothelial cells found in previous studies [17] are sufficiently large to assume any transport resistance to oxygen from the endothelium may be neglected [18].

Planes of symmetry were applied to the geometry where appropriate to reduce the size of the region required for simulation. For simulations in which the upper wall is exposed to environmental oxygen, a single, central plane of symmetry is applied while the sealed device allows for two planes of symmetry.

### 2.4. Parameterization

Parameters for the required physical properties of materials and OCR of cells and tissue taken from literature are listed in table 1.

**Table 1.** Simulation parameters from literature.

property	symbol	units	value	reference
viscosity of DMEM	*μ*_DMEM_	Pa . s	7.8 × 10^−4^	Bacabac *et al.* [14]
wall shear stress in perfusion vessel	WSS	Pa	0.5	Polacheck *et al.* [15]
atmospheric oxygen	*C*_0_	mol m^−3^	0.179	Al-Ani *et al.* [7]
diffusion coefficient of DMEM	*D*_DMEM_	m^2^ s^−1^	2.86 × 10^−9^	Al-Ani *et al.* [7]
diffusion coefficient of collagen I gel	*D*_col_	m^2^ s^−1^	2.15 × 10^−9^	Colom *et al.* [19]
diffusion coefficient of muscle tissue	*D*_musc_	m^2^ s^−1^	2 × 10^−9^	Davis *et al.* [20]
maximum OCR flux of endothelial cells	*J*_eth_	mol m^−2^ s^−1^	*ρ*_eth_ · *V*_eth_	
density of endothelial cell seeding	*ρ*_eth_	cells m^−2^	5 × 10^8^	Viñals *et al.* [21]
OCR of an endothelial cell	*V*_eth_	mol cell^−1^ s	8 × 10^−17^	Steinlechner-Maran *et al.* [22]^a^
Michaelis–Menten constant, endothelial cells	*k*_eth*M*_	mol m^−3^	5.5 × 10^−4^	Abaci *et al.* [23]
OCR of a muscle precursor cell	*V*_*mc*_	mol cell^−1^ s	2 × 10^−16^	Wagner *et al.* [24]
maximum OCR of muscle tissue	*V*_*M*Max_	mol cell^−1^ s	4.12 × 10^−3^	Davis *et al.* [20]
Michaelis–Menten constant, muscle tissue cells	*k*_MM_	mol m^−3^	1.33 × 10^−3^	Davis *et al.* [20]
OCR of a neuron	*V*_neur_	mol cell^−1^ s	8.13 × 10^−17^	McMurtrey [25]

^a^Mean of values in study.

Our *in vitro* experiments show endothelial cell cultures growth can constrict the radius of the perfusion vessel and so the perfusion vessel radius described here refers to the radius of the lumen of the cultured endothelial cells rather than the initial vessel radius. An initial media oxygen concentration, *C*_0_, was applied throughout the device at *t*_0_ and as a constant concentration over the inlets of perfusion vessels and the upper wall in simulations in which the bioreactor was exposed to atmospheric oxygen. Unless otherwise stated, *C*_0_ was set to 0.179 mol m^−3^, a concentration of oxygen in media exposed to air at sea level and 37°C [7]. A zero-flux boundary condition was applied to all additional exterior wall surfaces of the device.

Coefficients of oxygen diffusion for DMEM [7], collagen gel [19] and muscle tissue [20] taken from literature are shown in table 1. It was assumed that the presence of endothelial cells, muscle precursor cells and neuron cells do not significantly alter coefficients of oxygen diffusion of their respective substrate media. Values of cell OCR parameters for endothelial cells, muscle cells and muscle tissue, taken from literature, are also described in table 1. Previous studies show a range of values for the OCR of a single neuron. Ribiero *et al.* [26] show a basal rate of approximately 3.3 × 10^−17^ mol cell^−1^ s for mouse neurons *in vitro* and maximum rate of approximately 6.3 × 10^−17^ mol cell^−1^ s. Here, we apply a conservative value taken from McMurtrey *et al.* [25] of *V*_neur_ = 8.13 × 10^−17^ mol cell^−1^ s. The *in vivo* partial pressure of oxygen in tissue has been found to be around 40 mmHg, resulting in a tissue oxygen concentration of 0.052 mol m^−3^ [6]. We, therefore, set a 0.052 mol m^−3^ threshold for ‘normoxic’ tissue oxygen concentrations with which to compare against the oxygen concentrations at the centre of the muscle cavity. A concentration significantly above this threshold indicates hyperoxic conditions, while concentrations below tend to hypoxia and then oxygen deficit. As rates of cell proliferation and decay are diverse and dependent upon experimental variables, the simulations defined here assume cell populations to be in an equilibrium state and so do not include models of cell dynamics.

An unstructured tetrahedral mesh was applied in COMSOL using the *‘Physics-controlled’* method for defining mesh resolution. A mesh refinement study showed no significant variations in predicted oxygen concentrations or wall shear stresses between default *‘coarse’*, *‘normal’* and *‘fine’* COMSOL meshing regimes and so a coarse mesh was applied.

### 2.5. Simulations

To provide a value of oxygen concentration within the muscle cavity for comparison, the values of oxygen concentrations at mesh vertices lying on a two-dimensional horizontal cross-section of the muscle cavity were recorded and the mean calculated.

To assess the viability of the device, simulations were conducted on a sealed bioreactor in which oxygen flux is set to zero on external boundaries and on a bioreactor with the upper surface exposed to environmental oxygen. In both simulations, oxygen is perfused via DMEM flowing through two endothelial cell lined perfusion vessels with radius 100 μm, each spaced 1 mm laterally to a central cavity of radius 100 μm, containing muscle cells in a density of 3 × 10^7^ cell ml^−1^.

To determine the physical limitations of oxygen transport within the sealed device, simulations were conducted varying (i) concentrations of muscle precursor cells from 1 to 8 × 10^7^/ml, (ii) radius of the perfusion vessel from 20 to 200 μm, (iii) distance between perfusion vessel and muscle chamber centrelines from 0.8 to 2 mm and (iv) radius of muscle chamber from 100 to 250 μm. Simulations of the above physical variables were repeated using diffusion and OCR properties of mature muscle tissue.

For the more efficient bioreactor design comprised of two muscle chambers and a single endothelium-lined perfusion chamber, a study was performed to estimate the dimensions of bioreactors which will provide muscle tissue with *in vivo* oxygen concentrations (0.052 mol m^−3^) for any size of perfusion vessel radius (*r*_*p*_), muscle chamber radius (*r*_*m*_) and distance between chamber centres (*l*_*c*_). To achieve this, 16 different bioreactor designs were simulated with a range of physically realistic values of *r*_*p*_, *r*_*m*_ and *l*_*c*_. For each bioreactor simulation, mean oxygen concentration in the muscle chamber filled with mature muscle fibres was recorded. To define designs which provide normoxic conditions for muscle, sets of simulations were conducted in which one component (either *r*_*p*_, *r*_*m*_ or *l*_*c*_) was varied while the other two remained static. Quadratic regression was applied to the variable component and resulting mean oxygen concentrations in an iterative fashion until a bioreactor set-up was found producing oxygen concentrations of 0.052 ± 5 × 10^−4^ mol m^−3^. Surface fitting was applied in MATLAB to interpolate the component data for all 16 bioreactor designs. To ensure the interpolated surface was effective at predicting normoxic conditions and not overfitted to the data we applied first, second, third and fourth order surfaces and compared their predictions of *l*_*c*_ for five additional simulated bioreactor designs predicted to provide normoxic conditions. The root mean square deviation (RMSD) between interpolated and simulated values of *l*_*c*_ for each of the five additional bioreactor designs was calculated for each order of surface fit, with the closest match between interpolated and simulated results applied as the order of interpolation in the final model. The surface was then re-fitted to include the results for the five additional designs so that the final model was informed by the results of 21 simulated bioreactor designs. The equation of the surface provides a function for predicting which bioreactor set-ups will result in normoxic muscle conditions for *all* values of *r*_*p*_, *r*_*m*_ and *l*_*c*_.

Finally, the maximum sustainable density of neurons seeded within the bioreactor was estimated by varying neuron concentrations from 0.5 to 5 × 10^6^ cells ml^−1^.

## 3. Results

### 3.1. An enclosed bioreactor can provide a controllable oxygen supply for muscle growth

**Figure 2.**
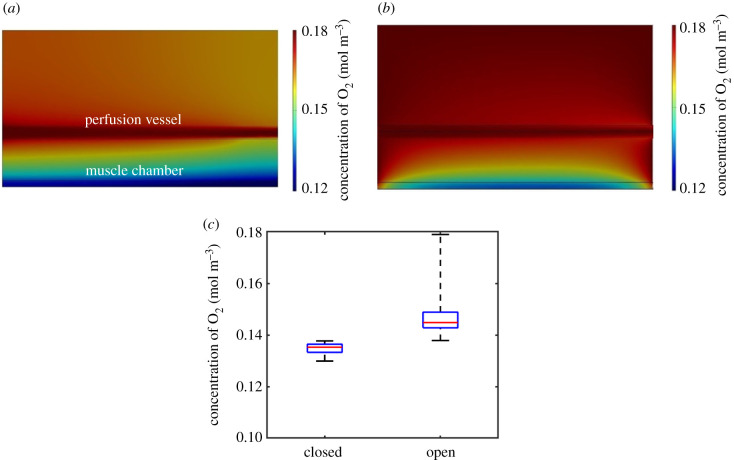
Comparison of oxygen transport in sealed and open devices. Oxygen concentrations in horizontal cross-sections of bioreactor in which (*a*) no oxygen flux is permitted through external boundaries and (*b*) upper boundary of device is exposed to atmospheric oxygen concentrations in air. Direction of flow in perfusion vessels is from right to left. (*c*) Boxplots of oxygen concentrations along horizontal cross-section of muscle tissue cavity for sealed and exposed devices.

Our simulations (figure 2*a*–*c*) show that a bioreactor with exterior walls which prevent oxygen flux allows a median oxygen concentration of 0.135 mol m^−3^ throughout the muscle cell cavity when perfused with DMEM through the two lateral channels. Though lower than that provided by a device open to environmental oxygen (0.145 mol m^−3^), it is significantly higher than the threshold normoxic oxygen concentration in tissue of 0.052 mol m^−3^. This shows that for muscle cells seeded at a reasonable density of 3 × 10^7^ cells ml^−1^, there is a substantial capacity within the device for additional oxygen consumption from the co-culturing of other cell types within the bioreactor before hypoxic conditions are reached. Additionally, the oxygen concentration profile along the muscle cell cavity (figure 2*a*,*b*) was shown to be more uniform in the sealed device. Uniformity of conditions ensures experimental outcomes are consistent and repeatable and allow full control of cell culture conditions via the media entering the perfusion vessel.

### 3.2. The proposed design is robust allowing flexibility in design and cell seeding volume

**Figure 3.**
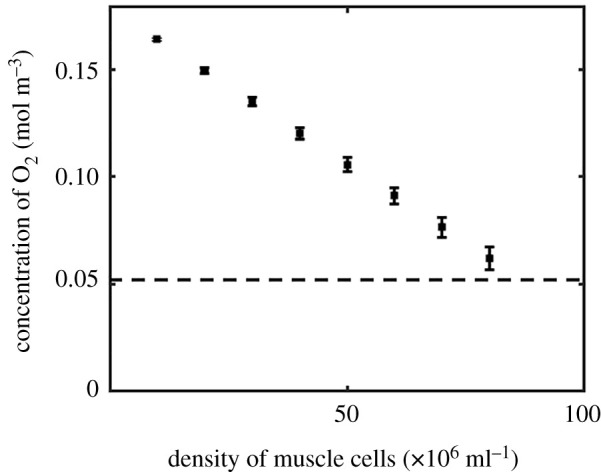
Oxygen transport to the muscle chamber with varying muscle cell density. Oxygen concentration along the horizontal cross-section of the muscle cavity when seeded with varying densities of muscle precursor cells. Dashed line represents estimated *normoxic* oxygen concentration.

Our simulations estimate that the sealed bioreactor described above can provide normoxic oxygen concentrations with muscle cells densities of $80-90\times 10^{6}\;{\mathrm{cells}\;\mathrm{ml}}^{-1}$ (figure 3*a*), well above the typical density seeded in culture experiments. This indicates that sufficient oxygen can be provided to initiate muscle cell seeding and initial growth and proliferation through media perfusion alone. The properties of the muscle chamber in the numerical model were then altered to simulate mature muscle tissue, with a coefficient of diffusion lower than that of the surrounding collagen I gel.

**Figure 4.**
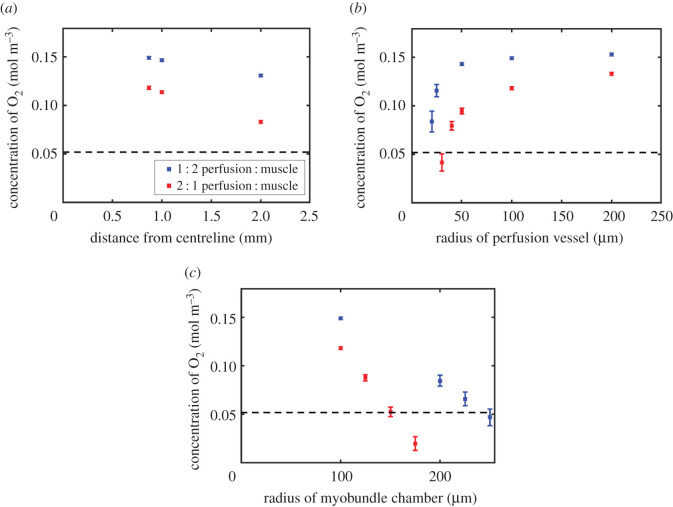
Oxygen transport limits of device for 1 : 2 and 2 : 1 perfusion vessel to muscle tissue chamber ratios. Oxygen concentration along the horizontal cross-section of the muscle cavity filled with mature myobundles tissue for devices with one perfusion vessel and two muscle chambers (blue) and two perfusion vessels and one muscle chamber (red). (*a*) Varying the distance between the muscle cavity and perfusion vessel, (*b*) varying the radius of the perfusion vessel and (*c*) varying the radius of the muscle tissue chamber.

Moving the perfusion vessel further from the muscle cavity was shown to provide sufficient oxygen for all distances within a 5 mm width unit (figure 4*a*). Varying the radius of the perfusion vessels between 200 and 50 μm was estimated to have a minor (approx. 0.01 mol m^−3^) effect on oxygen availability to skeletal muscle tissue (figure 4*b*) while radii less than 50 μm exhibited a dip in mean concentration of oxygen provided and an increase in variation suggesting they are unsuitable for culturing muscle tissue. The sealed bioreactor was shown to be able to sustain mature muscle tissue at *in vivo* oxygen concentrations in a cavity of radius 245 μm (figure 4*c*).

### 3.3. A more efficient arrangement of microtubes can sustain a larger volume of muscle tissue

Given the robust nature of the preliminary bioreactor design described above, we propose a more efficient configuration in which two muscle tissue chambers are set laterally to a single central perfusion vessel. Simulations indicate that this configuration can sustain muscle tissue above the normoxic *in vivo* (0.052 mol m^−3^) oxygen threshold. We predict that sufficient oxygen can be provided when the when the perfusion vessel radius is 35 μm or larger (figure 4*b*) and that a muscle chamber radius of up to 150 μm (figure 4*c*) can be sustained. Our simulations show that a sealed bioreactor with a central perfusion vessel of radius 100 μm can sustain two lateral muscle chambers of radius 150 μm with 0.052 mol m^−3^ of oxygen, giving a maximum muscle tissue volume of 7.06 × 10^−10^ m^3^ for the device. The same oxygen concentration in a device with two perfusion vessels and a single muscle chamber is achieved with a 245 μm radius muscle chamber, giving a maximum muscle tissue volume of 9.43 × 10^−10^ m^3^. While there is a gross increase in potential muscle volume in the double perfusion vessel configuration, the addition of the second vessel is costly and so a comparison of muscle tissue volume per vessel is more appropriate. The double perfusion vessel configuration, therefore, yields 4.71 × 10^−10^ m^3^ of tissue per vessel and so a 2 : 1 muscle chamber to perfusion vessel ratio results in a 50% increase in maximum muscle volume per perfusion vessel.

**Figure 5.**
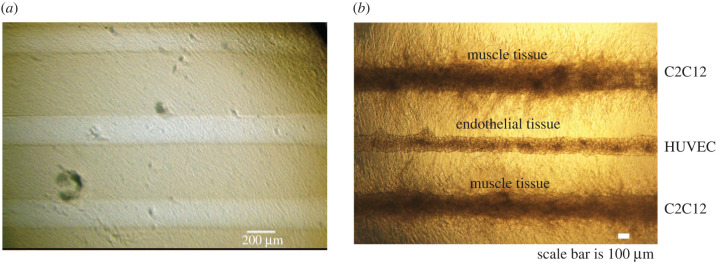
*In vitro* example of a bioreactor with 2 : 1 ratio of muscle chambers to endothelial lined perfusion vessel. Human umbilical vein endothelial cells (HUVECs) and C2C12 muscle cells successfully grown in a bioreactor with a more efficient ratio of muscle cells to perfusion chambers. The device illustrated here was encased in PDMS which is permeable to atmospheric oxygen and so represents an ‘open’ device rather than a ’sealed’ device and is shown here as a proof of design.

As a proof-of-concept to illustrate the design of the chip and show that muscle and endothelial cells can be co-cultured within a single device, a bioreactor with this more efficient chamber ratio was constructed, with the bioreactor encased in PDMS, and seeded with HUVEC and C2C12 mouse cells figure 5. It is important to note that since PDMS is permeable to oxygen this does not represent a sealed device. Both endothelial and muscle cells survived to maturity, indicating sufficient oxygen levels were provided.

### 3.4. A predictive model of bioreactor design for normoxic conditions

Having shown that a bioreactor with a 2 : 1 ratio of muscle chamber to perfusion vessel to be effective for a given set of reactor dimensions, we present a predictive model to determine the range of bioreactor configurations for which this improved set-up will provide normoxic conditions for muscle cells and tisuse. To achieve this, we conducted a simulation study of 21 bioreactors (16 for defining the model and five for validation) with different lengths of perfusion vessel radius (*r*_*p*_), muscle chamber radius (*r*_*m*_) and distance between chamber centres (*l*_*c*_). Using the dimensions of bioreactors with normoxic conditions as points and fitting a surface allows the creation of functions for predicting bioreactor design for any value of *r*_*p*_,*r*_*m*_ or *l*_*c*_. As well as a guide for conditions of normoxic muscle tissue, the fitted surface provides a design threshold beyond which conditions will be either increasingly hypoxic or hyperoxic depending upon if a component is lengthened or shortened. A study of the effects of the order of interpolation of the fitted surface (electronic supplementary material, table S1 and figure S1) showed that a surface of order 3 gave the lowest RMSD between interpolated and simulated bioreactor dimensions with normoxic conditions (28.2 μm s.e.±11.4 μm). Fitting a surface with order 4 produced a less effective fit due to over-fitting. A surface fit of order 3 was, therefore, applied to our final model. The resulting function is shown below arranged with the subject as radius of muscle chamber filled with mature muscle fibres (equation (3.1)), distance between centrelines of muscle and perfusion chambers (equation (3.2)) and radius of perfusion chamber providing WSS of 0.5 Pa (equation (3.3))

3.1 $$\begin{array}{rl} r_{m} & =128.2+1.768r_{p}-0.1242l_{c}-5.819\times 10^{-3}r_{p}^{2}\\ & \;-9.408\times 10^{-4}r_{p}l_{c}+1.033\times 10^{-4}l_{c}^{2}+1.296\times 10^{-5}r_{p}^{3}\\ & \;-1.488\times 10^{-9}r_{p}^{2}l_{c}+2.722\times 10^{-7}r_{p}l_{c}^{2}-2.873\times 10^{-8}l_{c}^{3} \end{array}$$

3.2 $$\begin{array}{rl} l_{c} & =1.476\times 10^{4}+48.01r_{p}-240.9r_{m}-0.2473r_{p}^{2}\\ & \;-5.808\times 10^{-2}r_{p}r_{m}+1.185r_{m}^{2}+4.072\times 10^{-4}r_{p}^{3}\\ & \;+3.731\times 10^{-4}r_{p}^{2}r_{m}-2.628\times 10^{-4}r_{p}r_{m}^{2}-1.852\times 10^{-3}r_{m}^{3} \end{array}$$

3.3 $$\begin{array}{rl} \mathrm{and}\;r_{p} & =-5305+78.791r_{m}+5.428l_{c}-0.3612r_{m}^{2}\\ & \;-6.359\times 10^{-2}r_{m}l_{c}-1.306\times 10^{-3}l_{c}^{2}+4.91\times 10^{-4}r_{m}^{3}\\ & \;+1.831\times 10^{-4}r_{m}^{2}l_{c}+8.488\times 10^{-6}r_{m}l_{c}^{2}+7.38\times 10^{-8}l_{c}^{3} \end{array}$$

**Figure 6.**
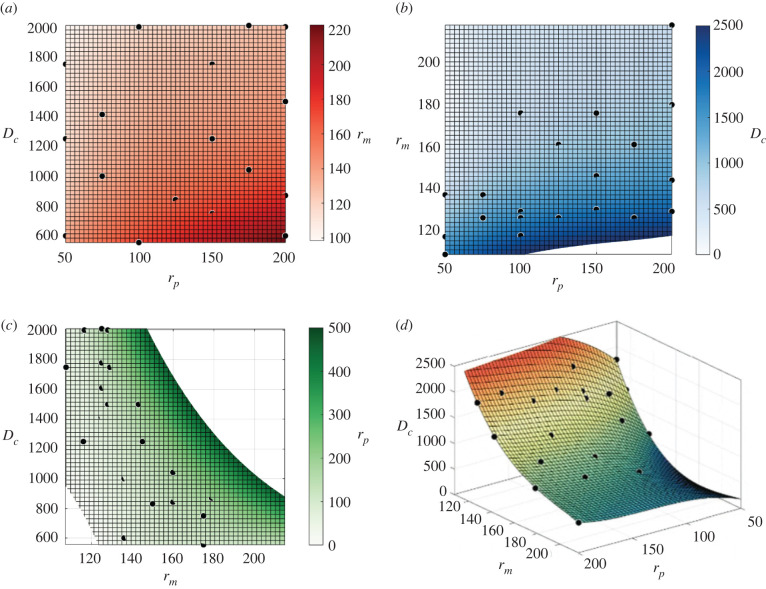
Predicted bioreactor dimensions producing normoxic conditions for muscle growth. Lengths of perfusion vessel radius (*r*_*p*_), muscle chamber radius (*r*_*m*_) and distance between centrelines of chambers (*l*_*c*_) for which mean oxygen concentration in a muscle chamber filled with mature myobundles is 0.052 mol m^−3^. (*a*) Visualization of equation (3.1) (*l*_*c*_ limited to maximum of 2500 μm), (*b*) visualization of equation (3.2), (*c*) visualization of equation (3.3) (*r*_*p*_ range limited from 0 to 500 μm). Black dots represent simulated bioreactors. All units are micrometres.

All length units are μm. Figure 6 shows graphical representations of functions (3.1)–(3.3). *r*_*p*_ is limited to a range from 0 to 500 μm, *l*_*c*_ from 0 to 5 mm and *r*_*m*_ from 0 to 250 μm.

### 3.5. A sealed device with two muscle chambers per perfusion vessel can generate *in vivo* oxygen conditions when co-cultured with motor neurons

**Figure 7.**
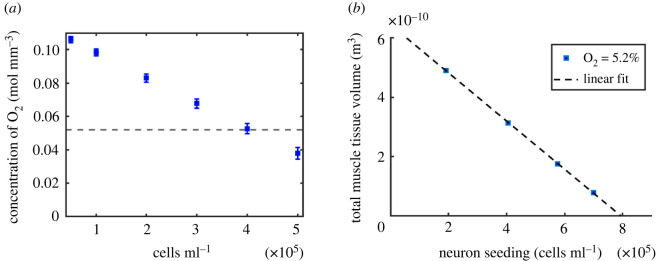
Oxygen transport to seeded neurons. (*a*) Oxygen concentration in 100 μm muscle tissue cavity at varying densities of seeded neurons (dashed line shows approximate *in vivo* tissue oxygen concentration). (*b*) Neuron cell densities and corresponding predicted muscle tissue volume required to provide oxygen concentration of 5.2% within the muscle chamber.

A sealed bioreactor with two muscle chambers of radius 100 μm, perfused by a central vessel of radius 100 μm was predicted to allow oxygen concentrations at normoxic levels or above when neurons at seeding densities up to 4 × 10^5^ cells ml^−1^ (figure 7*a*) are co-cultured within the bioreactor cavity. Estimates of the volume of muscle tissue required to sustain a normoxic oxygen level of 0.052 mol m^−3^ within the muscle cavity (figure 7*b*) show that the tissue volume reduces linearly with increasing neuron cell seeding density. Extrapolation of these simulated data predict that muscle tissue can be cultured at *in vivo* oxygen levels for neuron seedings below 8 × 10^5^ cells ml^−1^.

## 4. Discussion

The growth and behaviour of muscle cells and muscle tissue is dependent upon the oxygen environment [27], and so muscle-on-chip bioreactor design must allow control over the oxygen concentration reaching cells. Since the primary goal of muscle-on-chip technology is to recapitulate physiological muscle function, it is also necessary to ensure that tissue oxygen concentrations match *in vivo* conditions. Since atmospheric oxygen concentrations are significantly higher than those encountered *in vivo*, a bioreactor design which is permeable to oxygen would expose cells to hyperoxic conditions unless the entire chip is placed in a controlled atmosphere. A more practical solution is perfusion oxygen through a sealed device via flowing cell growth media.

Through simulation of oxygen transport in a proposed muscle-on-chip bioreactor comprised of a muscle tissue growth chamber and perfusion chamber lined with endothelial cells, we show that sufficient oxygen can be delivered in a sealed device by perfusion alone. As well as enabling control of the oxygen entering the system, we show that muscle tissue receives a more uniform distribution of oxygen when compared to a permeable device ensuring conditions for muscle cells are more homogeneous. The flow rate of media into the device was adjusted to create wall shear stresses sufficient to maintain endothelial cell function [15].

We describe an efficient bioreactor chamber configuration, applying a ratio of two muscle chambers to one perfusion vessel, to transport sufficient oxygen while maximizing the volume of muscle tissue produced. We extrapolate data from simulations of a range of bioreactor configurations to present a set of functions for calculating the bioreactor dimensions (diameters of the perfusion chamber, muscle growth chambers and the distance between chambers) predicted to allow normoxic conditions for muscle tissue growth. A sealed, perfused, device was shown to be capable of providing the normoxic conditions sufficient for sustaining a co-culture of motor neurons seeded throughout the bioreactor cavity.

Limited variation in oxygen solubility in commonly used cell culture media due to similarities in ionic strength and protein concentration [6] and high permeability of endothelial cells to oxygen suggest that these results can be applied to oxygen transport in bioreactor designs independently of media composition. Studying the transport of larger molecules may be important for device design but will be more sensitive to media composition and the permeability of the endothelial cell barrier.

We produced functions for calculating bioreactor designs to provide an *in vivo* oxygen concentration of 0.052 mol m^−3^. For simplicity we apply a single threshold to represent normoxia here, though Richardson *et al.* [27] show that intracellular oxygen partial pressures can range by ±6 mmHg (0.0078 mol m^−3^) in normoxic muscle tissue. Above the normoxia threshold, conditions are hyperoxic for cells. Hyperoxic conditions may be required if increasing cell yield is of greater importance than maintaining physiological conditions, or if the bioreactor is required to co-culture complementary cell species such as neurons. Conversely, devices with dimensions in which oxygen transport falls below this threshold will produce hypoxic conditions in which to study the behaviour of muscle tissue in a low oxygen environment [28]. A further application of these functions is the estimation of the diameter of myobundles required to provide normoxic conditions in a specific device configuration, providing a limit to the yield of the bioreactor.

## 5. Conclusion

Through FEM numerical modelling, we show that a muscle-on-chip bioreactor can provide a sufficient oxygen concentration to sustain muscle cell growth through perfusion of media alone. This would allow the device to be sealed from atmospheric oxygen and so provide greater control over the cell culture environment. We show that oxygenation via perfusion vessels is sufficiently robust to allow a ratio of one perfusion vessel to two muscle chambers for increased efficiency and tissue yield. We provide a model for predicting bioreactor dimensions which will allow muscle cells to experience normoxic conditions based upon fitting a surface to outputs from numerical simulations of multiple bioreactor configurations. We show that the perfused device is capable of sustaining neurons seeding throughout the cavity and predict the density of neurons which can be seeded in typical bioreactor configuration while still providing sufficient oxygen for muscle cell growth.

This study provides a guide for prospective designs of muscle-on-chip devices, particularly those with a view to co-culturing muscle cells with other cell species, allowing a targeted approach to creating a controlled oxygen environment for culturing muscle tissue.

## Data Availability

Data for dimensions of simulations of individual bioreactor configurations referenced in the text are provided in the file ‘Validation of surface.xlsx’. Electronic supplementary material is available online [29].
