# Correction to ‘Mathematical modelling of oxygen transport in a muscle-on-chip device’ (2022) by Hardman *et al.*

**DOI:** 10.1098/rsfs.2022.0057

**Published:** 2022-10-14

**Authors:** David Hardman, Manh-Louis Nguyen, Stéphanie Descroix, Miguel O. Bernabeu

*Interface Focus* **12**, 20220020. (Published online 12 August 2022). (https://doi.org/10.1098/rsfs.2022.0020)

The Funding section of the article ‘Mathematical modelling of oxygen transport in a muscle-on-chip device’, should read as follows:

This project has received funding from the European Union's Horizon 2020 research and innovation programme under grant agreement no. 801423. This research was funded in whole, or in part, by the Engineering and Physical Sciences Research Council (EPSRC) (grant nos. EP/R029598/1 and EP/T008806/1). For the purpose of open access, the author has applied a creative commons attribution (CC BY) licence to any author accepted manuscript version arising.

